# Over-Specification of Small Cardinalities in Referential Communication

**DOI:** 10.3389/fpsyg.2021.745230

**Published:** 2021-11-29

**Authors:** Natalia Zevakhina, Lena Pasalskaya, Alisa Chinkova

**Affiliations:** HSE University, Moscow, Russia

**Keywords:** over-specification, informativeness, referential communication, reference production, numerals, color adjectives

## Abstract

The paper presents experimental evidence for the over-specification of small cardinalities in referential communication. The first experiment shows that when presented with a small set (2, 3, or 4) of unique objects, the speaker includes a numeral denoting a small cardinality into the description of given objects, although it is over-informative for the hearer (e.g., “three stars”). On the contrary, when presented with a large set of unique objects, the speaker does not include a numeral denoting a large cardinality into their description, so she produces a bare plural (e.g., “stars”). The effect of small cardinalities resembles the effect of over-specifying color in referential communication, which has been extensively studied in recent years (cf. Tarenskeen et al., 2015; Rubio-Fernández, 2016, among many others). This suggests that, like color, small cardinalities are absolute and salient. The second experiment demonstrates that when presented with an identical small set of monochrome objects, the speaker over-specifies a small cardinality to a much greater extent than a color. This suggests that small cardinalities are even more salient than color. The third experiment reveals that when slides are flashed on the screen one by one, highlighted objects of small cardinalities are still over-specified. We argue that a plausible explanation for the salience of small cardinalities is a subitizing effect, which is the human capacity to instantaneously grasp small cardinalities.

## Introduction

It is well-acknowledged that sometimes speakers tend to convey more information than required. In doing so, they use redundant linguistic expressions. Imagine a situation where there is only one cup available in the visual context shared by the speaker and the addressee. In such a situation, the speaker utters (1), where she specifies the color of an object, even though the addressee can identify the object without mentioning its color. In other words, the speaker could produce (2) where she only names the object itself without specifying its color. Obviously, sentence (1) conveys more information than sentence (2). In Gricean terms (Grice, 1975), the former sentence is over-informative, whereas the latter one is minimally informative. Why did the speaker produce the over-informative sentence instead of uttering the minimally informative one?

(1) Give me the blue cup, please.
(2) Give me the cup, please.

There have been proposed two accounts for this puzzle: speaker-oriented and hearer-oriented. According to the speaker-oriented account, producing over-informative utterances requires less cognitive effort on the speaker’s part than producing minimally informative utterances (Pechmann, 1989). To illustrate, in a situation of describing an object that possesses several properties, the speaker does not need to think which properties are relevant to communicate. Rather, she produces those properties which have come to her mind, and she does not waste time and effort to make certain computations with respect to the discriminability. According to the hearer-oriented account, over-specification helps hearers identify relevant objects more rapidly, accurately and, therefore, more efficiently (Mangold and Pobel, 1988; Rubio-Fernández, 2016).

Some properties enable over-specification, whereas some others do not. Recent research on property redundancy in reference production has demonstrated that color is much more likely to be used redundantly than size, material, shape, pattern, location, and orientation (Mangold and Pobel, 1988; Belke and Meyer, 2002; Arts et al., 2011; Brown-Schmidt and Konopka, 2011; Gatt et al., 2013).

The contrast between color and other properties is accounted for in terms of absoluteness and salience (Belke and Meyer, 2002; Koolen et al., 2011; Tarenskeen et al., 2015 among others). The absoluteness of color means that color is not contingent upon the speaker, the addressee, a situation, etc. For instance, if the observer sees a red object, she does not have to take into consideration colors of surrounding objects, she can merely report “red.” On the contrary, size is relative since the degree of size of a given object is evaluated by the observer among the degrees of the size of surrounding objects. If a given object does not differ from surrounding objects in terms of size, size is not likely to be reported.

As for the salience, color is salient because of its high visual perceptibility. It is one of the features of pre-attentive analysis (Trick, 1992) and is computed early in visual processing (Livingstone and Hubel, 1988). Other properties (such as shape, material, pattern, or size) are not so salient and, therefore, are less likely to be reported.

It seems that both factors – absoluteness and salience – determine over-specification in reference communication. Take size. It is relative. Size is reported when it becomes contrastive and relevant for communication (see Van Gompel et al., 2014). In other words, it is specified when it is supposed to be salient. For instance, the visual context includes only one big plate, with the rest of the plates being small. However, it is unlikely to be reported when the visual context includes three big and three small objects of one type or three big and three small objects of various types: a plate, a cup, a spoon, etc. Moreover, the degree of the proportion between the objects does not seem to play a role here. To illustrate, Tarenskeen et al. (2015) manipulated size contrast between objects with a proportion 3:1 but did not receive a significant increase in over-specification of size. Now take pattern. It is absolute and salient. In this respect, it resembles color. However, Gatt et al. (2013) and Tarenskeen et al. (2015) showed that it is not as salient as color.

Focusing on color, it is noteworthy to highlight several factors that affect color over-specification. Firstly, color is more likely to be over-specified in polychrome contexts than in monochrome contexts (Belke and Meyer, 2002; Koolen et al., 2013; Rubio-Fernández, 2016). To illustrate, the speaker is more likely to say “Give me the blue cup, please” in a situation when she is presented with objects of different colors than in a situation when she is presented with objects with the same color, say blue. Bichrome (contrastive) contexts, which are a variety of polychrome contexts, but with a small set of two different colors, also facilitate color over-specification (see Tarenskeen et al., 2015). Secondly, color tends to be over-specified for atypically-colored objects in comparison to variably-colored or stereotypically-colored objects (Westerbeek et al., 2015; Rubio-Fernández, 2016). To illustrate, the probability that the speaker redundantly utters (3) with an atypically-colored wolf is higher than the probability that the speaker redundantly utters (4) with a variably-colored car, or (5) with a stereotypically-colored banana.

(3) Show me a purple wolf, please.
(4) Show me a red car, please.
(5) Show me a yellow banana, please.

Thirdly, color is more often over-specified when it is variable rather than stereotypical for a given category of objects (Sedivy, 2003; Rubio-Fernández, 2016). For example, the speaker would produce (4) to a higher degree than (5). Fourthly, color is more likely to be over-specified when referring to objects for which color is more important: e.g., artifacts like clothes or cars are more color-pertinent than geometric objects (Rubio-Fernández, 2016).

Going back to the distinction between color and size, Brown-Schmidt and Konopka (2011) argued that not only color but also number (or cardinality) is distinct from size. Color adjectives and numerals were reported more frequently and faster than size adjectives in reference communication. They were reported both in contrastive and non-contrastive contexts. On the contrary, size adjectives were reported significantly more often in contrastive contexts. Brown-Schmidt and Konopka (2011) suggested that the reason for this is that, unlike color and number, size is a context-dependent modifier. These findings accord with the following idea circulated in the literature: size is relative (context-dependent), whereas color is absolute (not context-dependent); size is non-salient, whereas color is salient. As for the number, according to the findings by Brown-Schmidt and Konopka (2011), it seems to be absolute and salient, like color. However, this fact was not directly addressed in the literature. Furthermore, it was not clear why number seems to be absolute and salient. Also, it was not clear which numbers were tested in Brown-Schmidt and Konopka (2011). Judging by Figure 2a–b in Brown-Schmidt and Konopka (2011: 308), the number, up to 5, was involved. On the whole, it seems that what is needed now is a more systematic study of number over-specification in referential communication. The present study addresses this issue.

### Subitizing and Salience of Small Cardinalities

For more than a century (since Bourdon, 1908), there has been acknowledged a fast, accurate, and confident apprehension of cardinalities of small sets (1–4 or even 1–8) which considerably decreases in cardinalities of large sets. To illustrate, when presented with three dots in a display, an observer undoubtedly and very rapidly determines the cardinality of the dots. This capability vanishes when the observer is presented with 15 dots in a display.

The phenomenon of immediately grasping the cardinality of few elements in a given set was coined as *subitizing* in Kaufman et al. (1949), with reference to Dr. Cornelina C. Coulter (Kaufman et al., 1949: 520), who suggested this term roughly meaning “sudden apprehension,” cf. also a similar term, *numerousness*, in Stevens (1938), Thomas et al. (1999), and Taves (1941). In Kaufman et al. (1949), subitizing was contrasted to *estimation*, that is, an approximate and less accurate apprehension of cardinalities of large sets, cf. also a close term *numerosity* in Stevens (1938), Thomas et al. (1999), and Taves (1941).

The question of what is the threshold for small sets is debated, but there has been a tacit agreement in the literature that the numbers from 1 to 3–4 belong to the subitizing range. The question whether the numbers ranging from 5 to 8 belong to the subitizing range is under discussion as well. The threshold seems to vary from person to person. It is also dependent on a particular experiment setting and some other factors (Jensen et al., 1950; Chi and Klahr, 1975; Atkinson et al., 1976; Akin and Chase, 1978; Oyama et al., 1981; Mandler and Shebo, 1982).

Importantly, subitizing is not the same as counting small cardinalities. Rather, it involves a separate cognitive mechanism (Revkin et al., 2008). Counting is effortful, error-prone and slow (Trick, 1992). It usually takes 250–350 ms per item. In contrast, subitizing is effortless, accurate, and rapid. It usually takes 40–100 ms per item. Subitizing has been argued to be a pre-attentive mechanism which allows an observer to grasp the cardinality of items without carefully counting them (Trick, 1992).

It seems natural to assume that the subitizing effect yields salience of small cardinalities in visual perception. Because of this, small cardinalities are expected to be reported in an over-informative environment similar to color, which has been argued to be a highly salient property in visual search and object identification.

### Absoluteness of Small Cardinalities

As shown above, color items are absolute. What about numerals? It is well-known that numerals might have two meanings: exact meanings “exactly n” and at-least meanings “at least n and possibly more” (see Papafragou and Musolino, 2003; Musolino, 2004; Breheny, 2008 among others). There has been a debate in the literature about which meaning is primary, and which one is secondary, as well as which contexts allow for which meaning of numerals. A standard view is that the at-least meaning is basic, and the exact meaning is modeled in Gricean terms as a scalar implicature. Be that as it may, to the best of our knowledge, the relation between absolute meanings of numerals and the subitizing effect has not yet been discussed. It seems reasonable to say that pre-attentively identifying a small cardinality, the speaker uses a numeral of a small cardinality in the exact meaning rather than in the at-least meaning. This idea is motivated by the fact that people subitize a numeric point rather than a numeric interval. If it is so, then subitizing yields the exact meanings of numerals, which are absolute (not relative). In terms of absoluteness, small cardinalities resemble color. Both types of properties are absolute.

### The Present Study

Relying upon what has been outlined above, in the present study we hypothesize that over-specification of small cardinalities and over-specification of color are significantly different from over-specification of large cardinalities (Hypothesis 1). Moreover, since color and small cardinalities are absolute and salient, if color is over-specified in reference production, small cardinalities are also expected to be produced over-informatively (Hypothesis 2). These two hypotheses are tested in the first experiment.

Before we move on, we need to draw a potential borderline between color and small cardinalities. As a reviewer pointed out, small cardinalities are a property of sets of objects, whereas color is a property of objects, such that a single set of objects could have different color objects, but not different cardinalities. Indeed, it is an important issue that should be addressed in further research. However, the experiments reported in this paper did not aim at testing implications that this difference might lead to. The present study is designed in such a way that participants have to describe a highlighted cell that displays either an individual object of some color, a set of objects of some cardinality, or a set of objects of some cardinality and color at the same time. Accordingly, the manner of displaying objects enables to ignore the difference between color and cardinalities in terms of their property status.

The first experiment uses visual contexts throughout all the conditions, in which color and cardinality are presented in a two-by-two contrastive way. Among four cells, two of them have either one identical color or cardinality, whereas two others have another identical color or cardinality. As reported in the literature (see the overview in the section “Introduction”), polychrome and bichrome contexts facilitate color over-specification, whereas monochrome contexts decrease it, mainly because monochrome contexts decrease color salience. It seems reasonable to assume that mono-cardinal contexts provide a similar reducing effect on cardinality over-specification. The question is what happens in non-contrastive contexts that are mono-cardinal and monochrome at the same time. The salience of color and small cardinalities enables to hypothesize that in non-contrastive visual contexts that include sets of objects with an identical cardinality and an identical color, if color is seldom over-specified, then small cardinalities are also supposed to be seldom over-specified (Hypothesis 3). This hypothesis is addressed in the second experiment.

Last but not least, on the supposition that the salience of small cardinalities is determined by subitizing, over-specification of small cardinalities presented in a flashed mode is expected to be similar to over-specification of small cardinalities presented in a non-speed mode (Hypothesis 4). The third experiment investigates this issue.

The materials of all three experiments are given in Appendix.

## First Experiment

### Participants

Ninety Russian native speakers voluntarily took part in the experiment (56 females, age range = 17–32, mean age = 21).

### Design

**FIGURE 1:**
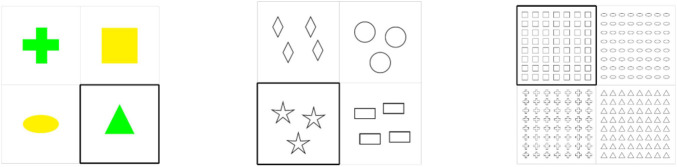
Examples of critical items used in the color, small cardinality, and large cardinality conditions in the first experiment.

The experiment has a between-subjects design. In order to verify Hypotheses 1 and 2, we created three conditions: Color, Small Cardinality and Large Cardinality conditions, see Figure 1. In all three conditions, we use 2 × 2 cells presented in one slide, each of which contains various geometric objects (squares, rectangles, crosses, circles, triangles, diamonds, stars, and ovals). The geometric objects are identical within each cell but are different among all cells. In all the conditions, one cell is a target and is highlighted, whereas the other three are distractors. A target cell took different positions throughout the experiment: it could be any of the 2 × 2 cells.

In the Color condition, two (out of four) cells comprise objects of one color, whereas two other cells comprise objects of another color. There are three colors: red, green, and yellow. In the Small Cardinality condition, two (out of four) cells include objects of one small cardinality, while two other cells include objects of another small cardinality. There are three small cardinalities: two, three and four. In the Large Cardinality condition, two (out of four) cells have objects of one large cardinality, whilst two other cells have objects of another large cardinality. There are three large cardinalities: 7 × 8 (56), 8 × 7 (56), and 8 × 8 (64). Each condition contains 48 critical items, that is, 8 geometric objects × 3 colors × 2 versions in the Color condition, 8 geometric objects × 3 small cardinalities × 2 versions in the Small Cardinality condition, 8 geometric objects × 3 large cardinalities × 2 versions in the Large Cardinality condition. Two versions of critical items in one condition differ only in two aspects: which cell is highlighted, and in which order the cells are displayed in a slide.

Depending on a condition, we expected that the participants would use the following ways of referring to objects. In the Color condition, there might be either a singular noun (e.g., “a square”) or a color adjective and a noun (e.g., “a red square”). In the Small Cardinality condition, the options are either a bare plural noun (e.g., “squares”) or a numeral and a plural noun (e.g., “two squares”). In the Large Cardinality condition, there might be either a bare plural noun (e.g., “squares”), a quantifier and a plural noun (e.g., “many squares”), or a numeral and a plural noun (e.g., “56 squares”). Importantly, a singular noun and a bare plural noun are minimal specifications of referring to given highlighted cells. A color adjective and a noun as well as a numeral and a noun are over-specifications. As for combinations of a quantifier and a noun, they do not seem to be minimal specifications. However, they do not seem to be over-specifications either, since adding information of a large amount of some set does not say anything about a cardinality of such a set, especially if other sets presented in a slide can also be referred to by means of the “many” expression. Therefore, it seemed reasonable to treat data with “many” (if they would occur) as minimal specifications.

**FIGURE 2:**
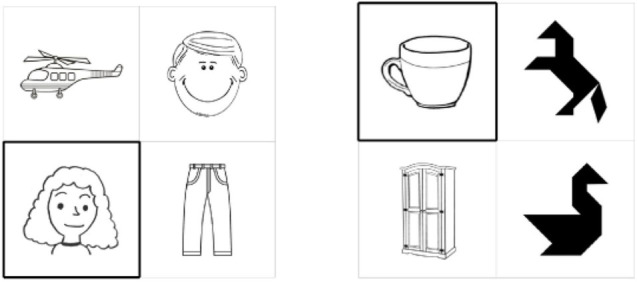
Examples of filler items in the first experiment.

Filler items were images of human faces, tangrams, and artifacts (crockery, furniture, transport, and clothes), cf. Figure 2. In parallel to critical items, each filler slide contains four images of two types: e.g., two artifacts and two tangrams, two human faces and two artifacts, two tangrams, and two human faces. There are 72 filler items that are identical in all the three conditions.

The idea behind using human faces as fillers was that their description involves higher participants’ concentration because they contain many features important to distinguish one face from another (see Koolen et al., 2013): with vs. without beard, with vs. without glasses, hair style, mood of a person, dress, etc. Tangrams seem to be even more difficult than human faces in reference production. On the contrary, artifacts are easier to be referred to, since their identification is effortless. All the fillers were intentionally left uncolored, that is black-and-white. The idea behind that was that participants were not supposed to concentrate on color of hair, dress, etc.

### Procedure

There were two versions of each condition with a random order of critical and filler items. Importantly, critical and filler items were counterbalanced, so that each pair of critical items was separated with at least one filler. Therefore, each condition formed two experimental lists. Each list had 120 items (48 critical items + 72 fillers). Each condition was presented for 30 participants (30 participants for a condition × 3 conditions = 90 participants). Before the experiment, participants were told that they had to describe a highlighted cell to a person who had the same set of cells but in a different order and who did not know which cell was highlighted. Participants’ task was to describe a highlighted cell to a person so that she understood which cell is referred to. After pressing the space key or the right arrow on the keyboard, participants moved from one slide to another one. In the instructions, they were asked to make sure that their interlocutor also changed their slide. Only when the interlocutor confirmed this, a participant could start describing the cell. This was done intentionally to provide participants with some time to carefully examine a given slide. There were a few practice trials (identical to fillers) before the experiment. Because of Covid-19, the experiment was conducted online, *via* Zoom. Participants gave permission to be audio-recorded. The participants were told that no correct answers are expected. They were instructed not to think too long but not to rush.

### Results

4,320 responses (48 critical items × 30 participants × 3 conditions) were received (1,440 responses for each condition). However, some of them were excluded due to participants’ metaphorical naming of objects, mostly in the Color condition (e.g., “yellow oval” was described as an antispasmodic pill). Out of 1,440 responses in the Color condition, 49 responses (3.4%) were excluded. Out of 1,440 responses in the Small Cardinality condition, 4 responses (0.28%) were excluded. Out of 1,440 responses in the Large Cardinality condition, 22 responses (1.5%) were excluded. Therefore, 1,391 responses for the Color condition, 1,436 responses for the Small Cardinality condition, and 1,418 responses for the Large Cardinality condition were used. Among them, 1,085 responses (out of 1,391; 78%) were over-specified in the Color condition, 1,347 responses (out of 1,436; 94%) included over-specification in the Small Cardinality condition, and 69 responses (out of 1,418; 4.9%) were over-specified in the Large Cardinality condition.

In the Large Cardinality condition, 308 responses (out of 1,440; 21%) which specified a large amount of objects without a numeral (e.g., “many ovals”) were treated on a par with bare plurals (e.g., “ovals”). In the Small Cardinality condition, 2 responses (out of 1,440; 0.14%) which specified a small amount of objects without a numeral (e.g., “some ovals”) were treated on a par with bare plurals (e.g., “ovals”).

**FIGURE 3:**
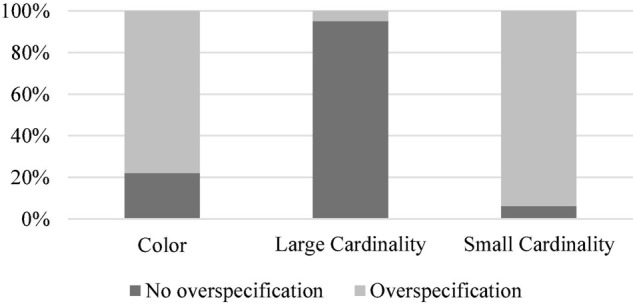
Distribution of (over-)specification in the color, small cardinality, and large cardinality conditions in the first experiment.

The results of the first experiment are visualized in Figure 3.

Using the R (R Core Team, 2020), we performed Kruskal–Wallis rank sum test and Wilcoxon rank sum test. The Kruskal–Wallis rank sum test indicated the main effect of Modifier (Color vs. Small Cardinality vs. Large Cardinality): *H* (2) = 2642.2, *p* < 0.0001. Multiple pairwise comparisons using the Wilcoxon rank sum test showed further differences between the conditions: Color vs. Large Cardinality (*p* < 0.0001), Color vs. Small Cardinality (*p* < 0.0001), and Small Cardinality vs. Large Cardinality (*p* < 0.0001).

### Discussion

The results of the first experiment confirm Hypothesis 1. They demonstrate that small cardinalities and color are over-specified in reference production to a greater extent than large cardinalities. In this respect, small cardinalities and color resemble each other. A plausible reason for this resemblance might be their absoluteness and salience. However, they are over-specified quite differently, with the proportions for small cardinalities being higher than the proportions for color. This only partially confirms Hypothesis 2. Also, in our view, this is an unexpected finding in the literature of over-specification in reference production that has mostly concentrated on color and that has tentatively concluded that it is the most over-specified property.

There arises a question as to what degree over-specification of small cardinalities is different from over-specification of color. Suppose there are a few cells with unique objects but of the same cardinality and color. Would small cardinality and color be over-specified in a similar way? This issue is addressed in the second experiment.

## Second Experiment

### Participants

Thirty Russian native speakers voluntarily participated in the experiment (20 females, age range = 20–26, mean age = 21). None of them took part in the first experiment.

### Design and Procedure

**FIGURE 4:**
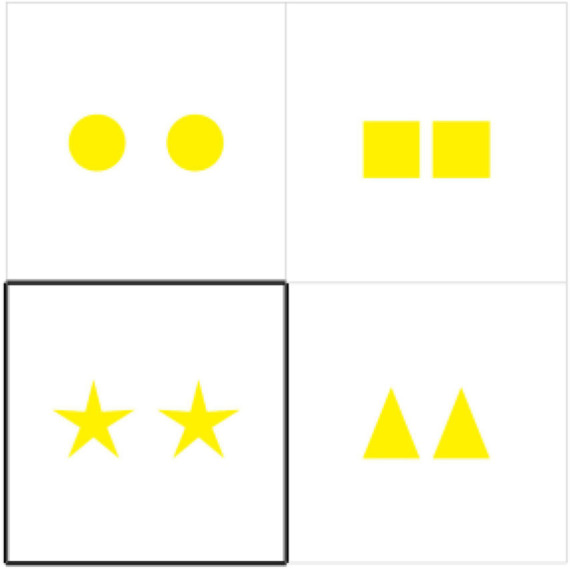
Example of a critical item used in the second experiment.

The design and procedure of the second experiment are similar to the design and procedure of the first experiment. We use 2 × 2 cells presented in one slide, each of which contains various geometric objects (squares, circles, triangles, and stars). The geometric objects are identical within each cell but are different among all cells. One cell of a slide is a target and is highlighted, whereas the other three are distractors. A target cell took different positions throughout the experiment: it could be any of the 2 × 2 cells. All the four cells comprise 4 different types of objects of one color and one cardinality. There are four colors (red, green, yellow, and blue) and three cardinalities (two, three, and four). This yields 48 critical items, that is, 4 geometric objects × 4 colors × 3 cardinalities. An example of a critical item is given in Figure 4.

We expected that the participants would use the following ways of referring to objects. There might be either a plural noun (e.g., “stars”); a numeral and a plural noun (e.g., “two stars”); a color adjective and a plural noun (e.g., “yellow stars”); or a numeral, a color adjective and a plural noun (e.g., “two yellow stars”).

72 fillers are identical to the fillers used in the first experiment.

Participants were presented with 120 items (48 critical items + 72 fillers), which were randomized. Before the experiment, participants were told that they had to describe a highlighted cell to a person who had the same set of cells but in a different order and who did not know which cell was highlighted. Participants’ task was to describe a highlighted cell to a person so that she understood which cell is referred to. After pressing the space key or the right arrow on the keyboard, participants moved from one slide to another. In the instructions, they were asked to make sure that their interlocutor also changed their slide. Only when the interlocutor confirmed this, could a participant start describing the cell. This was done intentionally to provide participants with some time to carefully examine a given slide. There were a few practice trials (identical to fillers) before the experiment. The experiment was conducted partially face-to-face and partially online (*via* Zoom) because of the Covid-19 pandemic: 6 vs. 24 participants, respectively. Participants gave permission to be audio-recorded. The participants were told that no correct answers are expected. They were instructed not to think too long but not to rush.

### Results

**FIGURE 5:**
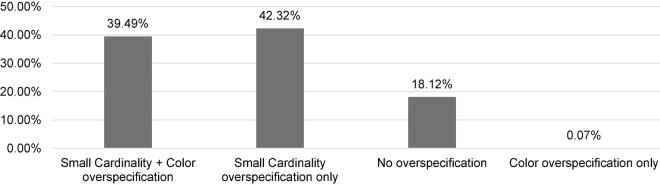
Distribution of small cardinality and color (over-)specification in the second experiment.

1,440 responses (48 critical items x 30 participants) were received. Among them, 60 responses (4.17%) were excluded since they were metaphorical expressions (e.g., “three stars” were called a three-star hotel). Out of 1,380 responses which were retained, only 250 responses (18.12%) were minimally informative expressions. 1,130 responses (81.88%) were over-informative either in color or in small cardinality. Among them, 584 responses (42.32%) were over-informative in small cardinality only, 545 responses (39.49%) were over-informative in both small cardinality and color, whereas 1 response was over-informative in color (0.07%). The distribution of responses is given in Figure 5.

Using the R (R Core Team, 2020), we carried out McNemar’s test. It showed that numerals were over-specified to a significantly higher degree than color adjectives (McNemar’s *χ^2^* = 579.02, *df* = 1, *p* < 0.0001).

Given the small proportion of participants who replied face-to-face, it does not seem reliable to say that the mode of presenting the materials to the participants yields differences in the data obtained. 18 out of the 24 participants who replied *via* Zoom (75%) used over-specifications of small cardinalities and/or color in more than 50% of their responses. Among them, 11 people (46%) over-specified both small cardinalities and color in more than 50% of their responses. Also, 7 people (29%) over-specified small cardinalities in more than 50% of their responses. As for those 6 participants who replied face-to-face, 2 of them used over-specifications of small cardinalities and color in more than 50% of their responses, and 4 used over-specifications of small cardinalities only. No over-specification occurred only in the responses of 6 out of 24 participants who replied *via* Zoom (25%), and color overspecification occurred in 1 response of 1 participant who replied *via* Zoom as well. Overall, it is questionable whether we can draw any solid conclusions from these observations.

### Discussion

The results of the second experiment disconfirm Hypothesis 3. They show that small cardinalities are not seldom and are over-specified to a greater extent than color. A plausible reason might be that small cardinalities are more salient than color and that their salience is determined by the subitizing effect. We verify this line of reasoning in the third experiment, where the slides are presented in a flashed mode.

## Third Experiment

### Participants

Thirty-one Russian native speakers voluntarily took part in the experiment (23 females, age range = 20–30, mean age = 24). None of them took part either in the first or in the second experiment.

### Design and Procedure

**FIGURE 6:**
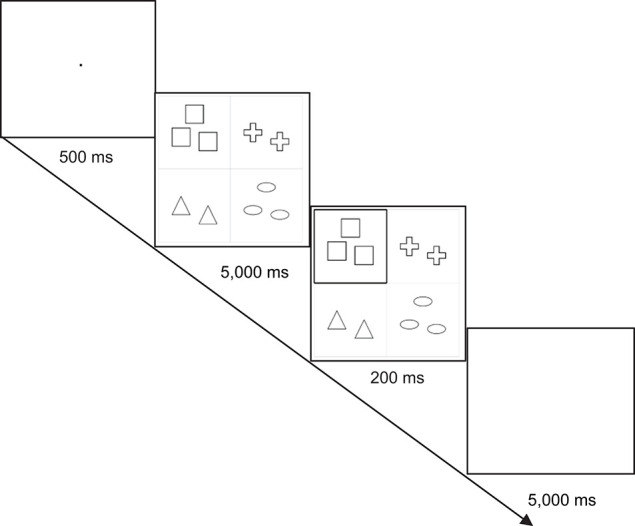
Example of the presentation sequence of a critical item in the third experiment.

Both critical and filler items are identical to the items used in the Small Cardinality condition of the first experiment (in total, 48 critical items + 72 filler items = 120 slides). However, the procedure was different. The slides for the Small Cardinality condition of the first experiment were presented in a flashed mode on the screen, cf. Figure 6. Firstly, participants saw a blank slide with a fixation dot for 500 ms. It was followed by a slide with four cells. Each cell contained a unique set of geometric objects. The cardinalities of two cells were identical and the cardinalities of the two other cells were also identical. No cell was highlighted. Such a slide appeared on the screen for 5,000 ms. During this time interval, participants could carefully examine a given slide. After that, participants were presented with the same slide, however, importantly, one of the cells was highlighted. The presentation of such a slide was for a quite short time, only for 200 ms. The reason for this short time interval was that, according to Trick (1992), subitizing usually takes 40–100 ms per item, that is on average 70 ms per item. Since one slide might include 2, 3, or 4 items in a highlighted cell, subitizing of 2 items in a cell is supposed to take 80–200 ms (on average 140 ms), subitizing of 3 items in a cell is supposed to take 120–300 ms (on average 210 ms), and subitizing of 4 items in cell is supposed to take 160–400 ms (on average 280 ms). Therefore, the interval would be enough or even shorter than enough to subitize the cardinality in the highlighted cell. A slide presented for 200 ms was followed by a blank slide appearing on the screen for 5,000 ms. During this slide, participants had to describe the highlighted cell of the previous slide. The 5,000 ms time frame to describe the cardinality of a highlighted cell seems to be long enough, since before the flashed 200 ms slide, the participants were presented with a slide of 5,000 ms used for careful examination of the objects given in a slide. In total, 480 slides were used.

Participants were instructed to get ready while being presented with the first slide, to carefully examine four cells in the second slide, to catch sight of which cell was highlighted in the third slide, and to describe the highlighted cell while being presented with the fourth slide. Participants had to describe highlighted cells to a person who had the same set of cells but in a different order and who did not know which cell was highlighted. That is, instead of a video presentation, a person had a mere presentation (as in the first experiment). There were a few practice trials (identical to fillers) before the experiment. Because of Covid-19, the experiment was held online, *via* Zoom. Participants gave permission to be audio-recorded. The participants were told that there were no correct answers. They were instructed not to think too long but not to rush. The video presentation lasted 22 min.

### Results

1,488 responses (48 critical items × 31 participants) were received. However, 90 responses (6.05%) of them were excluded because of the problems similar to those that occurred in the first experiment: participants’ self-corrections in counting objects (e.g., “three… four stars”), self-corrections in specifying cardinalities (e.g., “squares… three squares”), and errors in counting objects (e.g., “five ovals” when a cell with four ovals was highlighted). Interestingly, there was no metaphorical naming of objects. Moreover, there were some technical problems (unstable Internet connection) while presenting the materials to the participants *via* Zoom. This fact disqualified the recording of 400 audio responses to some of the critical items. The retained 1,398 responses were used. Among them, 1,281 responses (92%) included over-specification.

**FIGURE 7:**
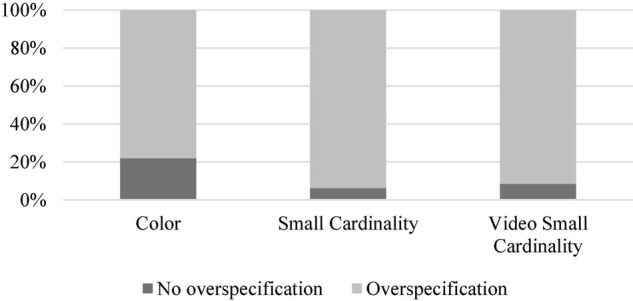
Distribution of (over-)specification in the color, small cardinality, and video small cardinality conditions (experiments 1 and 2).

The results of Experiments 1 and 2 are visualized in Figure 7.

Using the R (R Core Team, 2020), we conducted Kruskal-Wallis rank sum test and Wilcoxon rank sum test. The Kruskal–Wallis rank sum test showed the main effect of Condition (Color vs. Small Cardinality vs. Video Small Cardinality) in Experiments 1 and 2: *H* (2) = 193.17, *p* < 0.0001. Multiple pairwise comparisons using the Wilcoxon rank sum test revealed further differences between the conditions: Color vs. Video Small Cardinality (*p* < 0.0001), Small Cardinality vs. Video Small Cardinality (*p* < 0.026), and Color vs. Small Cardinality (*p* < 0.0001), with the latter difference being the result of the first experiment.

### Discussion

The results of the third experiment do not confirm Hypothesis 4. They demonstrate a significant difference between the Video Small Cardinality condition vs. the Small Cardinality condition (in the first experiment). Plausible reasons for this might lie in the mode of presentation. Firstly, the experiment contains too many slides (480). Secondly, the timing of 200 ms for the presentation of a slide with a highlighted cell seems to be relatively short, even though the previous slide is shown for 5,000 ms. Thirdly, the fourth slide in each 4-slide sequence has a time constraint of 5,000 ms. This might be not long enough to describe a flashed 200 ms slide. Be that as it may, the proportion of over-specification of small cardinalities in the third experiment is still relatively high. This suggests that subitizing plays a role in over-specifying small cardinalities, making them salient.

## General Discussion

The three experiments reported in this paper demonstrate that small cardinalities (up to 4) are over-specified in reference production because of the two factors: absoluteness and salience. In this respect, small cardinalities resemble color, which has been argued in the literature to be absolute and salient. As the results of the experiments show, small cardinalities are even more salient than color. A possible explanation for the salience and absoluteness of small cardinalities is subitizing (Kaufman et al., 1949 among many other studies). Subitizing makes small cardinalities salient and forces the corresponding numerals to be used in exact meanings (“exactly n”). The present study supports the idea that both absoluteness and salience play a crucial role in over-specification of cognitive domains in reference production.

## Data Availability Statement

The raw data supporting the conclusions of this article will be made available by the authors, without undue reservation.

## Ethics Statement

The studies involving human participants were reviewed and approved by the Institutional Review Board of the National Research University Higher School of Economics. The patients/participants provided their written informed consent to participate in this study.

## Author Contributions

NZ ran the statistical analyses and wrote the manuscript. LP created the stimuli and fillers and conducted the pilot studies. AC collected the data of the second experiment. All authors contributed to the article and approved the submitted version.

## Conflict of Interest

The authors declare that the research was conducted in the absence of any commercial or financial relationships that could be construed as a potential conflict of interest.

## Publisher’s Note

All claims expressed in this article are solely those of the authors and do not necessarily represent those of their affiliated organizations, or those of the publisher, the editors and the reviewers. Any product that may be evaluated in this article, or claim that may be made by its manufacturer, is not guaranteed or endorsed by the publisher.

## Appendix

The materials of the three experiments reported in the manuscript are available *via* the link: https://osf.io/xfrj7/.
